# Perioperative focused cardiac ultrasound: a brief report

**Published:** 2021-02-16

**Authors:** Amish Sheth, Anahita Dabo-Trubelja

**Affiliations:** 1 Department of Anesthesiology and Critical Care, Memorial Sloan Kettering Cancer Center, USA; 2 Department of Anesthesiology and Critical Care, Director, Perioperative Echocardiography and Ultrasound Director, Onco-Anesthesia Fellowship, Memorial Sloan Kettering Cancer Center, USA

## Abstract

**Background:**

The anesthesiologist’s emerging role as a perioperative physician has challenged the field to broaden its scope of practice to meet the demands of the patient undergoing surgery today. This brief report aims to identify the indications, clinical impact on management decisions, and perioperative focused cardiac ultrasound accuracy in patients scheduled for non-cardiac surgery.

**Methods:**

A review from the Department of Anesthesia Perioperative Echocardiography database on transthoracic echocardiography was performed, including clinical, demographic, indications, therapeutic impact, and accuracy from February 1, 2017 to October 10, 2019.

**Results:**

A total of 220 FoCUS exams were identified. FoCUS was performed in 55% males and 45% females. The average age was 66.5 years, and 68% of patients were designated ASA 3 classification. The majority underwent thoracic procedures with a history of cardiovascular disease for hemodynamic instability in the post-anesthesia care unit (PACU). In this group, 94% had a change in management. New findings in 9 patients resulted in pre-induction management change. FoCUS was also performed intraoperatively to differentiated hemodynamic instability, significantly altering care. Postoperatively, new wall motion abnormalities findings escalated care to the cardiology service. Immediate assessment of hemodynamic instability altered care and postoperative recovery location in a significant number of patients. In all cases, FoCUS was used to guide management in the differential diagnosis of the acute event and to assess treatment response.

**Conclusion:**

This review demonstrates that FoCUS is an excellent clinical adjunct in the perioperative period. Diagnostic accuracy and efficiency by pattern recognition helped answer clinically significant questions and guide management. The non-invasive approach of POCUS and its rapid adaptation makes it an exciting area of future research.

## Introduction

Focused transthoracic cardiac ultrasound (FoCUS) is a critical diagnostic tool in the acute care setting, allowing clinicians to rapidly assess cardiac function in the context of the patient’s clinical condition. FoCUS uses ultrasound as an adjunct to recognize specific ultrasound signs that suggest a narrower list of potential diagnoses in specific clinical settings.^1^ Perioperative cardiac focused ultrasound is generally performed on two main groups of patients: a) Those at elevated risk for cardiac or hemodynamic compromise are screened for pathology prior to induction of anesthesia and may alter pre-induction management in terms of fluids, vasopressors, and invasive monitoring and b) those who experience cardiac or hemodynamic compromise after induction of anesthesia or at any time in the post-anesthesia care unit (PACU). The utility of FoCUS performed by anesthesiologists significantly impacts the clinical management of patients scheduled for non- cardiac surgery.^2^ One study found that significant clinical findings on FoCUS were confirmed by follow-up formal transthoracic echocardiography in 52 out of 57 patients (91%) and that no severe aortic stenosis, severe ventricular dysfunction, or significant pericardial effusion were missed.^3^ This brief report aims to identify the indications, clinical impact on management decisions, and accuracy of perioperative focused cardiac ultrasound in patients scheduled for non-cardiac surgery by anesthesiologists with expertise in critical care echocardiography.

## Methods

Institutional review board approval #18–407 was obtained with waiver of consent. A review from the Department of Anesthesia Perioperative Echocardiography database on transthoracic echocardiography was performed from February 1, 2017, to October 10, 2019. Data collected included clinical, demographic, indications, therapeutic impact, and accuracy of echocardiographic studies. All patients had undergone non-cardiac cancer surgery at our tertiary cancer center, where all cancer surgeries are performed, precluding cardiovascular surgeries. Focused cardiac echocardiography was performed and interpreted by an anesthesiologist with expertise in critical care echocardiography or both transesophageal and transthoracic echocardiography at the request of an anesthesiologist assigned to the case. In all cases, the American Society of Echocardiography 2013 guidelines for performing focused cardiac echocardiography were followed. Indications included global assessment of biventricular function, wall motion abnormalities suggestive of ischemia, gross valvular abnormalities, pericardial effusion, hemodynamic assessment, arrhythmias, EKG changes/chest pain, and cardiac arrest.

**Figure 1:**
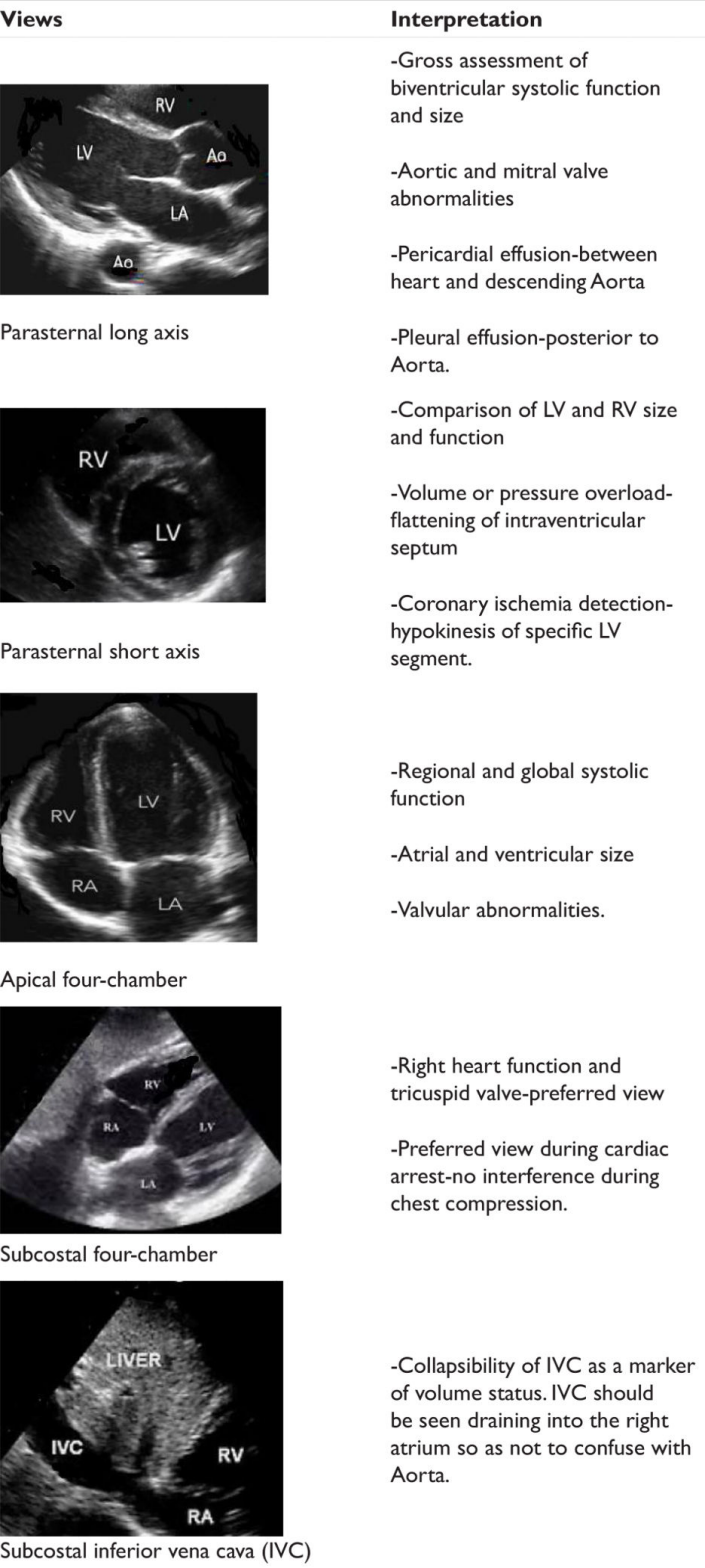
Focused Cardiac Ultrasound Views and Possible Interpretations. LV, Left ventricle; RV, right ventricle; RA, right atrium; LA, left atrium; Ao, aorta; IVC, inferior vena cava.

A focused cardiac echocardiogram was performed with the Philips infinity system using a S5– 1MHz broadband pure wave sector array transducer. Standard parasternal, apical, and subcostal windows acquired four heart views plus the subcostal inferior vena cava window as outlined in Figure 1. Qualitative and quantitative assessments were obtained depending on the indication and windows available in each patient. General estimation of left and right ventricular size and function were made, and all four cardiac valves were examined. The IVC (inferior vena cava) was observed for collapsibility and estimation of right atrial (RA) pressure. Pericardial and pleural effusions were noted.

A formal report incorporated patient demographics, date, place, and indication for FoCUS. Also, surgical procedure, hemodynamic assessment, significant echocardiographic findings, management changes influenced by FoCUS, cancellation of cases, and escalation of care to the cardiology consult service were compiled. Hemodynamic assessment was classified according to the 2015 American Society of Echocardiography guidelines for using echocardiography for therapeutic intervention in adults.

## Results

**Figure 2:**
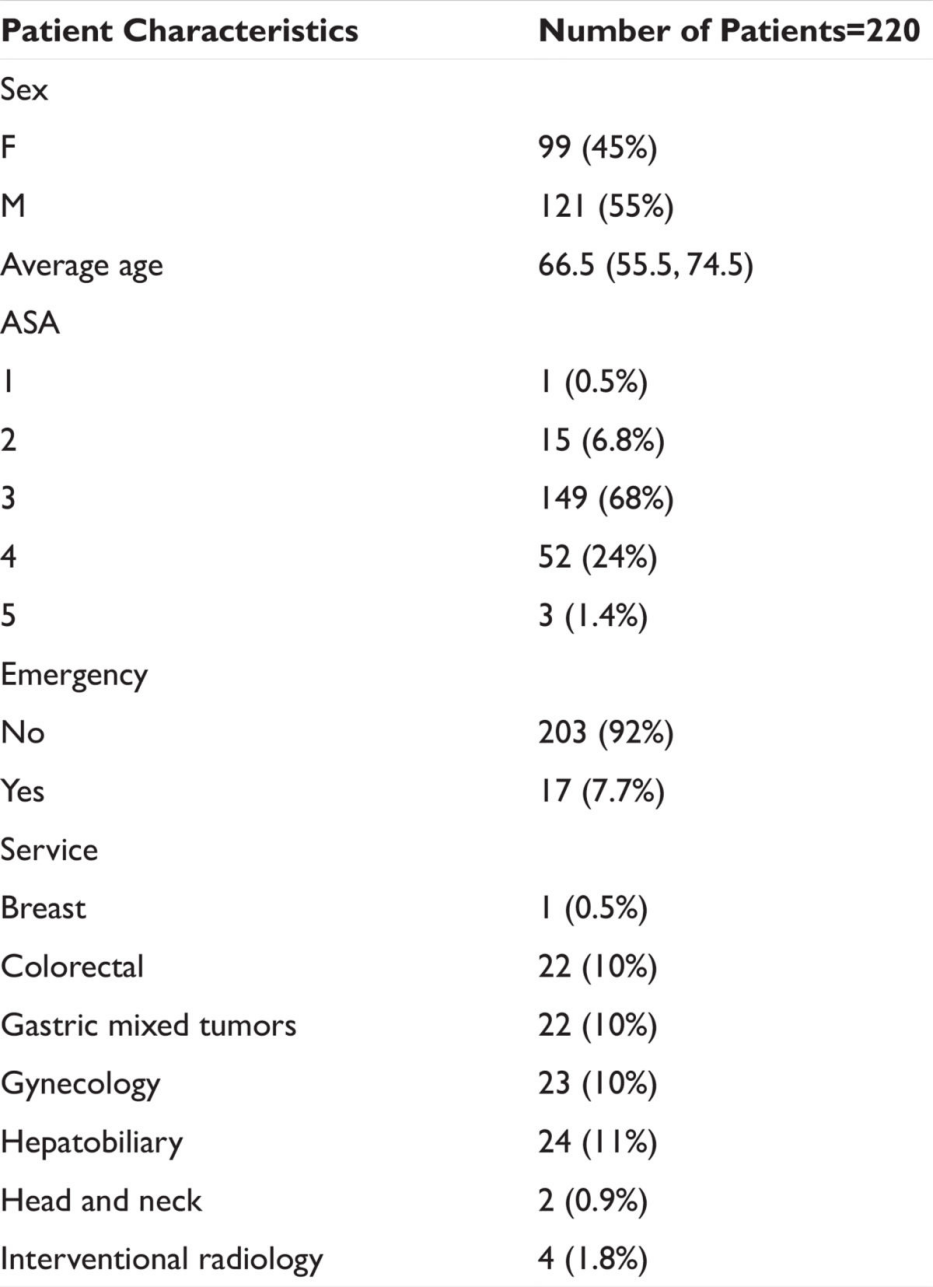

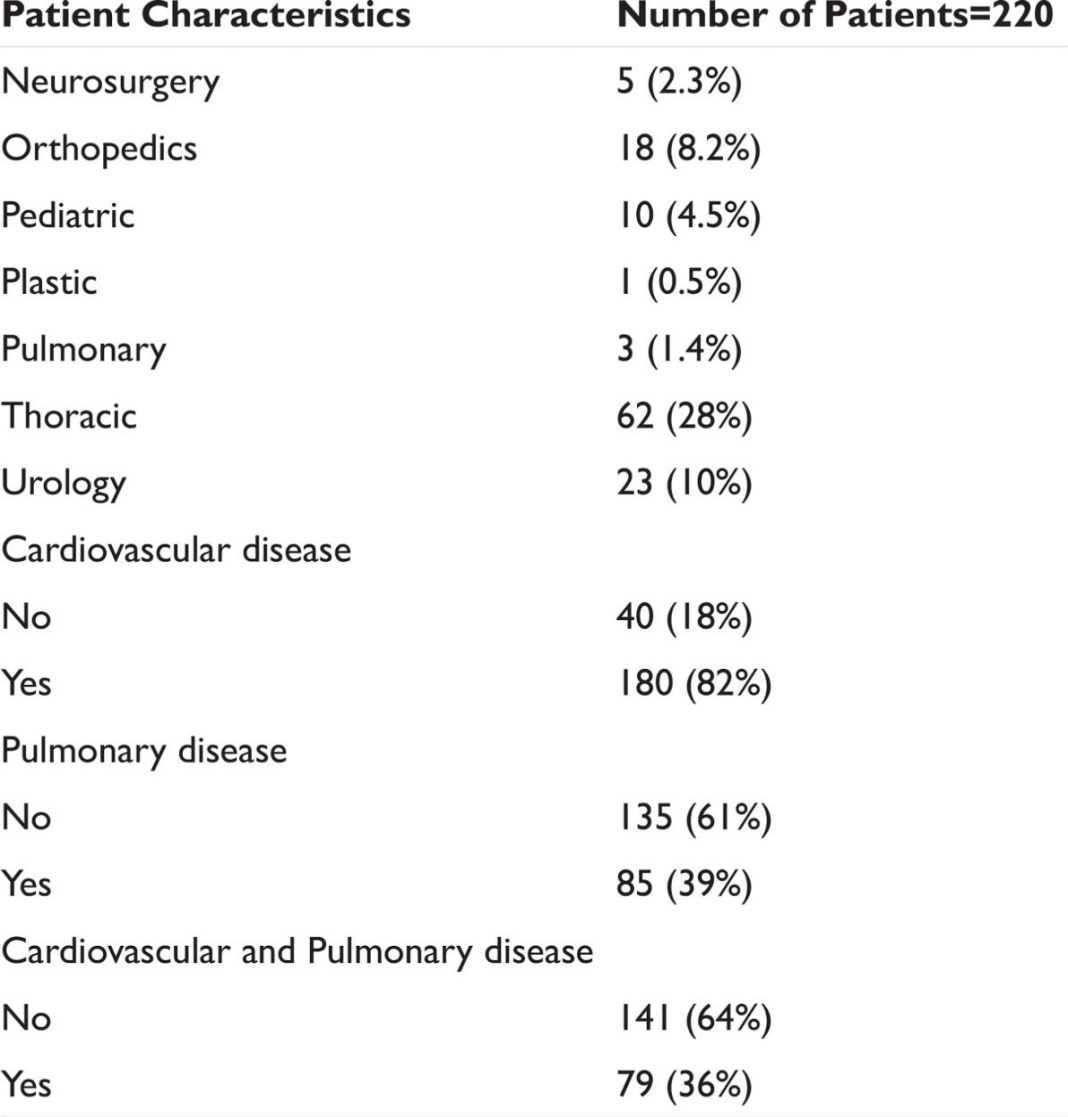
Patient Demographics and Characteristics.

**Figure 3:**
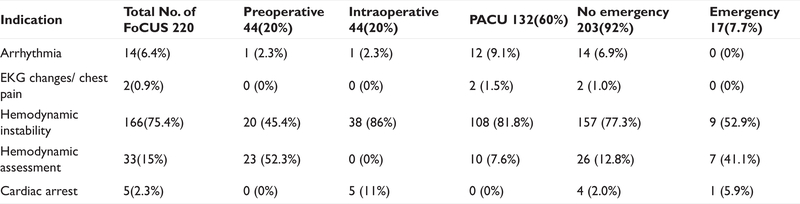
Perioperative location where FoCUS was performed, indications and emergency status.

A total of 220 focused cardiac echocardiography studies were identified from the Department of Anesthesia Perioperative Echocardiography database on transthoracic echocardiography from February 1, 2017, to October 10, 2019. The use of FoCUS aimed to identify specific indications in the perioperative setting, which would answer a specific question and immediately impact the patient’s perioperative management. Primary demographics and patient characteristics are outlined in Figure 2. FoCUS was performed in 55% of males and 45% of females with an average age of 66.5 years. Although a wide distribution of ASA classification was represented, most patients, 149/220 or 68%, fell into the ASA 3 classification. FoCUS examinations were performed in 62/220 (28%) of the patients undergoing thoracic procedures followed by major abdominal surgery equally distributed between colorectal, hepatobiliary, and gastric mixed tumors. Among demographics, the pediatric population above 18 years was also included. At our institution, a diagnosis that falls within pediatric cancer is treated by the pediatric service even if the patient is above 18 years of age. A history of cardiovascular disease was the most common comorbidity observed (180/220, 82%), followed by a history of pulmonary disease in 85/220 (39%) of patients, and both comorbidities were present in 79/220 (36%) of patients. Figure 3 details the location where the FoCUS examination was performed and the indications. A more significant percentage of FoCUS examinations, 132/220 (60%), occurred in the post-anesthesia care unit (PACU) and equally between the preoperative and postoperative phase of care area. The majority of examinations (81%) performed were for hemodynamic instability and occurred in the PACU. There was no significant relationship between indication and emergency status (p-value 0.2), i.e., no significant difference in indication based on emergency status. This could be because there were only 17 emergency cases-a small sample size.

**Figure 4:**
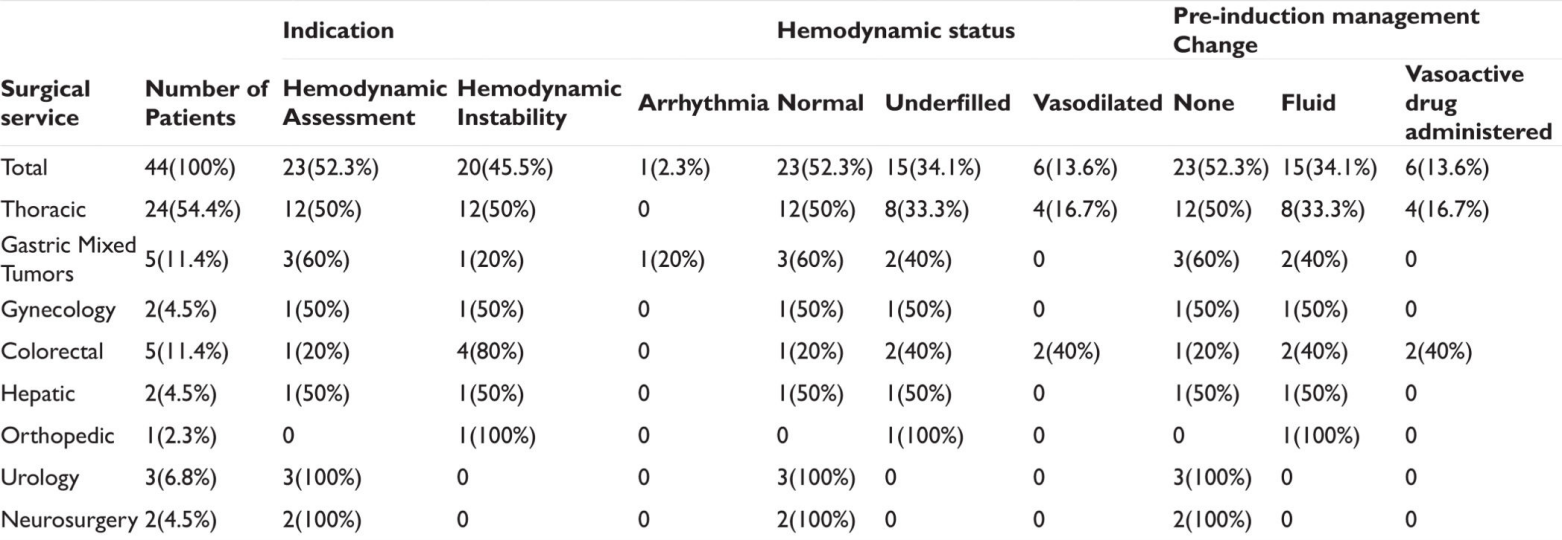
Pre-induction focused transthoracic cardiac ultrasound performed in 44 patients (values are the number of patients and percentage).

Pre-induction FoCUS assessment was performed in 44/220 (20%) patients (Figure 4). The majority of possible cardiac abnormality suspicion was not previously appreciated based on the primary anesthesiologist’s preoperative interview. Normal findings were found in 23/44 (52.3%) patients, and there was no change in the induction of general anesthesia. New findings were identified in 9/44 (20.5%) patients. One patient had unexplained tachycardia; FoCUS examination revealed total collapse of the left lung by a massive pleural effusion with mediastinal shift to the right. FoCUS significantly altered the pre-induction course. In this patient, a chest tube was placed under local anesthesia to remove pleural fluid and an arterial line before general anesthesia. Eight patients had a new pericardial effusion with no hemodynamic compromise. In consultation with cardiology and surgery, it was decided that surgery would proceed. Pre-induction invasive blood pressure monitoring was placed. The patients were followed in the postoperative period by the cardiology consult service. Changes in fluid management were also common. Assessment for unexpected hemodynamic instability and arrhythmia occurred in 21/44 (47.8%) patients. There were 15/44(34.1%) patients who received a fluid bolus. In 6/44(13.6%), extra fluid was avoided, and a vasoactive drug and invasive blood pressure monitoring were initiated.

**Figure 5:**
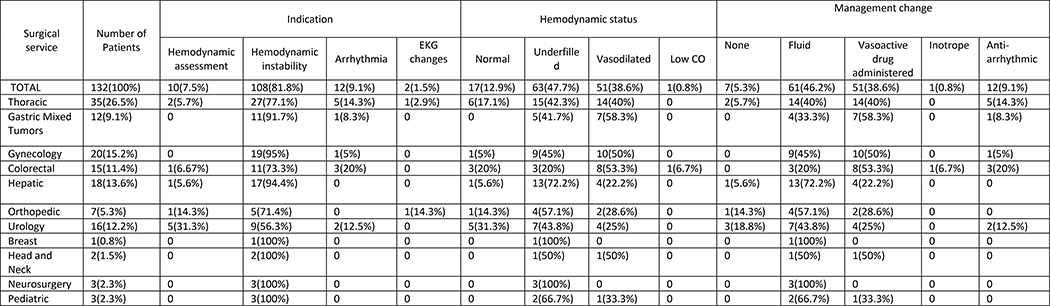
Intraoperative focused transthoracic cardiac ultrasound performed in 44 patients (values are the number of patients and percentage).

**Figure 6.**
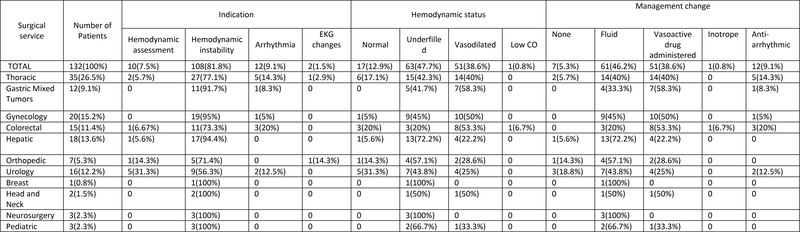
Postoperative focused transthoracic cardiac ultrasound performed in 132 patients (values are number of patients and percentage)

Intraoperative FoCUS was performed in 44/220 (20%) patients (Figure 5). The echo team was consulted to identify the differential diagnosis of hemodynamic instability in 38/44 (86.4%) patients. In this group, 40% received additional fluid boluses, 40% a vasoactive medication, and three patients were administered an inotrope for a low cardiac output state. FoCUS was performed in five patients during cardiac arrest differentiating cardiac standstill in two patients with unsuccessful resuscitative efforts and pulseless electrical activity in three patients with full recovery. One patient developed atrial fibrillation. FoCUS was unremarkable and was comparable to a comprehensive TTE performed in the PACU. A postoperative CT scan revealed a pulmonary embolus. In the PACU, postoperative FoCUS was performed on 132/220 (60%) patients (Figure 6). The majority 108/132(81.8%) was performed for hemodynamic instability, and 94% had altered care.

Hemodynamic status was classified as normal, underfilled, vasodilated, or a low cardiac output state. In 43/51(15%) patients, regional anesthesia performed for postoperative care contributed to vasodilation, and a vasoactive substance was administered. The remaining eight patients from this group required a change in the recovery location, necessitating ICU level care. Two patients developed sepsis. The one patient with a low cardiac output state on FoCUS was administered an inotrope. The patient suffered a myocardial infarction and underwent emergent catheterization. In the underfilled group of 63 /132(47.7%) patients, all except 2 received a fluid bolus after initial FoCUS evaluation. These two patients were young, healthy, with good urine output on ERAS protocols, who did well. Three patients did not respond to initial fluid boluses and were transferred to the ICU for further management and care. There were 10/132(7.5%) patients assessed with FoCUS in the setting of pre-existing coronary artery disease, with clinical and perfusion evidence of exercise-induced myocardial ischemia and angina; five patients were escalated to the cardiology consult service for possible new wall motion abnormalities. New-onset arrhythmia occurred in 12/132 (9.1%) patients, FoCUS was performed to guide initial management, and further care was escalated to the cardiology service. Acute chest pain/EKG changes occurred in 2/132 (1.5%) patients. FoCUS showed possible new wall motion abnormalities. The high suspicion for a cardiac event prompted escalation to the cardiology consult service for further management and care with one patient undergoing emergency catheterization for acute myocardial infarction.

## Discussion

FoCUS, in the hands of an anesthesiologist, is a useful tool in the perioperative setting. As an adjunct to physical examination, FoCUS can be used to identify significant ventricular dysfunction, significant valvular pathology and assist in estimating intravascular volume status. It can identify life-threatening cardiac causes of shock such as pericardial tamponade, acute cor pulmonale, hyperdynamic left ventricular (LV) outflow obstruction due to hypovolemia, the use of inotropes, acute severe valvular failure, severe LV and right ventricular (RV) dysfunction. In cardiopulmonary resuscitation, FoCUS differentiates cardiac standstill from pulseless electrical activity(PEA) and guides management beyond the ACLS algorithm.^4^ In this series, a significant number of patients had some alteration of their perioperative care based on initial information obtained from FoCUS. FoCUS was feasible to perform and with diagnostic findings comparable to traditional echocardiography interpreted by cardiologists. Previous studies have suggested FoCUS examinations may help in the management of perioperative patients.^5^ The most common indication for a FoCUS exam in this series was hemodynamic instability in patients with pre-existing cardiovascular disease undergoing thoracic surgery and performed in the PACU. A more significant proportion of thoracic patients were on ERAS protocol with regional anesthesia, and FoCUS answered the diagnostic dilemma where extra fluid was avoided in those with ventricular dysfunction. The use of FoCUS made a significant change in management in our patient population. When faced with unexplained hemodynamic instability, FoCUS can serve as an adjunct to the clinical examination and rapidly provide point of care diagnostic information. FoCUS was useful for identification and exclusion of significant underlying pathology, as well as for improved monitoring and assessment of therapeutic response. Perioperative unexplained hemodynamic instability is a class one indication for transesophageal echocardiography in the non- cardiac patient. The patient may be similarly assessed non-invasively with transthoracic echocardiography, and FoCUS may serve as an alternative to invasive monitoring in managing the unstable non-cardiac patient at any time during the perioperative course.^6^

In our group of high-risk cardiac patients, ultrasound examination of the heart was used as a screening tool before anesthesia induction. This in no way should replace a formal cardiology evaluation in high-risk cardiac patients before surgery. In our patient population, FoCUS was comparable to formal echocardiographic findings. Also, we had four patients with large anterior mediastinal masses where FoCUS as a screening tool identified a small underfilled LV, changing the pre-induction course. There were also nine new findings on FoCUS not previously appreciated on formal preoperative echocardiography. This may be due to the timing of preoperative echocardiography assessment in our cancer population, in the setting of chemotherapy treatment, and the necessity to undergo surgery. The information derived from a FoCUS exam was used to support hemodynamic optimization and, in some cases, altered the induction plan altogether. Information derived helped guide management decisions regarding invasive vascular access and maintenance of anesthesia in a manner consistent with previously reported results.^7^

A focused assessment of patients with pre-existing cardiac disease or new onset of chest pain in the postoperative period may predict adverse cardiac events.^8^ Evaluation of chest pain with echocardiography to assess for wall motion abnormality has been used in the emergency department with success.^9^ TEE has shown to be a better predictor of wall motion abnormality than EKG changes in the intraoperative setting.^10^ Applied in the postoperative period, evaluation of wall motion abnormalities may lead to a more rapid diagnosis of reversible ischemia and institution of targeted treatment. Given the frequency with which ischemia was ruled out in our patient population, chest pain in the PACU appears to be an area of underutilization of the FoCUS examinations. In this series, FoCUS promptly assessed new wall motion abnormalities and ruled out a cardiac event in one patient. This expedited cardiology consults. FoCUS was found to correlate with formal transthoracic echocardiography performed by the cardiology service.

FoCUS allows visualization throughout the entire perioperative period, including preoperative evaluation, intraoperative hemodynamic monitoring, and postoperative evaluation of cardiopulmonary status.^11^ The FoCUS exam is feasible and can be performed rapidly. The views obtained are adequate to assess hemodynamic status despite limited acoustic windows and challenging intraoperative patient positioning. In the postoperative setting, mechanical ventilation as well as surgical dressings impede a full FoCUS evaluation. Sometimes the only views obtained are the parasternal long and short axis. Semiquantitative assessment of MAPSE and TAPSE can be performed and is a fair assessment of left and right ventricular longitudinal function.^12^

The usefulness of FoCUS in cardiac arrest in non-cardiac surgery has been well documented. The etiology of cardiac arrest can be established with FoCUS, making it paramount in managing the patient beyond ACLS. Definite therapy can be quickly instituted in a life-threatening situation.^13^ Our echoes performed during cardiac arrest provided valuable information, differentiated cardiac standstill vs. PEA, ventricular systolic and diastolic function, as well as intravascular volume status assessment. The perioperative cancer environment is different from the ICU and the emergency department regarding commonly seen pathology. The cancer patient has additional risks related to chemotherapy, radiation therapy, and immunotherapy induced end-organ damage. Theoretically, FoCUS evaluation in the preoperative setting can reveal unexpected findings such as cardiac compression by tumors, pericardial and pleural effusions, and unexpected treatment- related cardiomyopathies. These findings allow an alteration in risk stratification, which can influence anesthesia management, change monitoring techniques, encompass more invasive vascular access, and determine post-recovery unit admission. Despite the increasing evidence for the usefulness of FoCUS in the perioperative period, this modality is underutilized by anesthesiologists. There is no formal recommendation for training, evaluation, or certification within the field of anesthesiology, except the anesthesiologist intensivist, which may account for the lack of incorporation into daily anesthetic practice.^14^ Although we have identified several indications for the use of FoCUS, the most common indication remains hemodynamic assessment, most commonly performed in patients with pre-existing cardiac disease undergoing thoracic procedures. The limited, focused cardiac ultrasound exam is reliable and comparable to formal studies.^15^ While it may help guide management, its effect on outcomes remains unclear.

## Conclusion

In the perioperative period, FoCUS allows for the anesthesiologist as a perioperative physician to check for coexisting diagnosis, categorize shock, respiratory failure, and ongoing effects of therapeutic treatments. Real-time physiologic data reflect dynamic changes in response to medical therapies and follow the evolution of critical illness by serial examinations, allowing for the integration of FoCUS findings into a complete management plan. In the hands of the anesthesiologist, FoCUS should exist beyond the cardiovascular evaluation. Evaluation of lung, abdominal, and vascular structures in combination with echocardiography may prove useful in diagnosing and managing the complex cancer patient and may even change medical care in the perioperative period. It is therefore essential to consider this area in future research. The challenge may come in numbers to power the treatment effect. Obtaining a sufficient sample size will be challenging and denying the use of FoCUS to guide management may not be an option.
